# ‘Nur'turing tumor T cell tolerance and exhaustion: novel function for Nuclear Receptor Nur77 in immunity

**DOI:** 10.1002/eji.202048869

**Published:** 2020-10-25

**Authors:** Sanne C. Lith, Bram W. van Os, Tom T.P. Seijkens, Carlie J.M. de Vries

**Affiliations:** ^1^ Department of Medical Biochemistry Amsterdam UMC, Amsterdam Cardiovascular Sciences Institute for Infection and Immunity Amsterdam The Netherlands; ^2^ Department of Medical Biochemistry Amsterdam UMC, Amsterdam Cardiovascular Sciences Cancer Center Amsterdam Amsterdam The Netherlands; ^3^ Department of Internal Medicine Department of Hematology Amsterdam UMC, Vrije Universiteit Amsterdam The Netherlands

**Keywords:** immunotherapy, nuclear receptor, NR4A1, thymocyte selection, T cell tolerance

## Abstract

The nuclear receptor Nur77 is expressed in a multitude of tissues, regulating cell differentiation and homeostasis. Dysregulation of Nur77 signaling is associated with cancer, cardiovascular disease, and disorders of the CNS. The role of Nur77 in T cells has been studied for almost 30 years now. There is a clear appreciation that Nur77 is crucial for apoptosis of self‐reactive T cells. However, the regulation and function of Nur77 in mature T cells remains largely unclear. In an exciting development, Nur77 has been recently demonstrated to impinge on cancer immunotherapy involving chimeric antigen receptor (CAR) T cells and tumor infiltrating lymphocytes (TILs). These studies indicated that Nur77 deficiency reduced T cell tolerance and exhaustion, thus raising the effectiveness of immune therapy in mice. Based on these novel insights, it may be proposed that regulation of Nur77 activity holds promise for innovative drug development in the field of cellular immunotherapy in cancer. In this review, we therefore summarize the role of Nur77 in T cell selection and maturation; and further develop the idea of targeting its activity in these cells as a potential strategy to augment current cancer immunotherapy treatments.

## Graphical abstract

Nur77 expression and activation in T cells promotes tolerance and exhaustion. Tolerance comprises negative selection, suppression of self‐reactive T cells by Tregs, and T cells pushed into anergy. Exhaustion involves expression and activation of inhibitory receptors, such as PD‐1 and TIM‐3, resulting in lower effector cytokine release whilst promoting tumor growth.

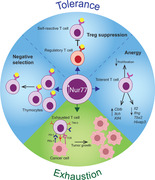

## Introduction on Nur77

Nur77 (also known as NR4A1, NGFI‐B, TR3, or NAK) is a transcription factor belonging to the “NR4A” subfamily of the human nuclear hormone receptor superfamily. This subfamily comprises Nur77 in addition to Nurr1 (NR4A2) and NOR‐1 (NR4A3). The NR4As perform tissue‐specific roles in processes such as DNA repair, metabolism, tumorigenesis, and inflammation with variable redundancy [1, 2, 3, 4]. Nuclear receptors bind DNA through a homologous, highly conserved DNA‐binding domain (DBD) composed of two zinc fingers. The N‐terminal trans‐activation domain (TAD) varies most in length and sequence among nuclear receptors, whereas the ligand‐binding domain (LBD) at the C‐terminus has a typical structure composed of 12 α‐helices [5]. The NR4A receptors bind DNA as monomers on a canonical target sequence known as the NGFI‐B‐response element, or as homodimers that recognize the Nur‐responsive element. Moreover, Nur77 and Nurr1 can also heterodimerize with the retinoid X receptor (RXR), mediating transcriptional response by binding a direct repeat element spaced by five nucleotides (DR5) [6, 7]. At present, the exact protein surfaces involved in dimerization have not been explored for Nur77.

For most nuclear receptors, transcriptional activity is mainly regulated by binding of a specific ligand to the so‐called ligand‐binding pocket in the LBD, followed by a conformational change and subsequent recruitment of cofactors to initiate target‐gene transcription[8]. In contrast, the LBD of NR4As seemingly lacks such a ligand‐binding pocket, because the potential pocket region is packed with hydrophobic bulky side chains of amino acids [8]. The few agonists that have been reported to modulate Nur77 activity thus far, bind the surface of the LBD and show low specificity [9, 10, 11]. The N‐terminal TAD of Nur77 plays an important role in its transcriptional activation and is instrumental for the recruitment of cofactors [12]. Like other nuclear receptors, Nur77 undergoes posttranslational modification among which phosphorylation, which influences its transcriptional activity and cellular localization.

The recent discovery that Nur77 plays a crucial role in T cell tolerance and exhaustion in cancer will be described first in this review. To improve our understanding of the underlying mechanism of Nur77 action in different types of T cells, we will summarize the wealth of knowledge obtained over the years on the expression, regulation of activity, and function of this nuclear receptor in thymocytes and mature T cells. The old and more recent data will be placed in broader perspective and development of novel cancer treatment strategies will be discussed.

## Nur77 in T cell tolerance and exhaustion in cancer

Two independent studies have recently identified NR4A members as critical mediators of T cell dysfunction in solid tumors [13, 14]. One of the recent studies focused on tumor‐infiltrating lymphocytes (TILs) and the second study investigated the role of Nur77 in chimeric antigen receptor (CAR) T cells. In the tumor microenvironment, T cells are constantly exposed to tumor‐antigens, which often give rise to exhausted TILs. These exhausted cells express a specific set of so‐called tolerance‐inducing genes, accompanied by self‐tolerance and reduced release of effector molecules [15, 16]. In this situation, programmed death protein‐1 (PD‐1) receptor on T cells is activated through the binding to its ligand (PDL‐1), expressed by the tumor cells. PD‐1 downstream signaling prevents proliferation and cytotoxicity, also known as “T cell anergy” [17]. Other T cell inhibitory surface receptors such as LAG‐3, CD160, 2B4, TIM‐3, BTLA, and CTLA‐4 similarly mediate T cell anergy [18].

Liu et al. [13] performed a genome‐wide epigenetic and gene expression screen using an in vitro T cell tolerance induction system with mouse cells. In tolerant T cells (Ttol), epigenetic signatures showed Nur77 expression to be increased, together with anergy‐related genes (*Cblb, Dgka, Rnf149, Tagap1*, and *Nfatc1)*, whereas effector genes (*Il2, Ifng, Gzmb, Il17a*, *and Il21)* were downregulated. Transcriptome analysis of *in vivo*‐generated Ttol cells also showed an upregulation of Nur77 and a reduction in expression of effector genes, such as *Il2, Ifng*, and *Tbx21*. In line with these observations, overexpression of Nur77 in CD4^+^ T cells led to a strong downregulation of effector genes (*Il2, Ifng, Tbx21*, and *Hivep3)*, while upregulating anergy‐related genes (*Cblb, Itch*, and *Klf4*). Furthermore, Liu et al. [13] showed that Nur77 deficiency suppressed T cell tolerance in a peptide‐induced tolerance model, whereas IL‐2 expression was induced. Regarding the CD8^+^ T cell dysfunction we described earlier, mice with OVA‐expressing EL4 cells (E.G7 lymphoma) showed that upon transfer of Nur77‐deficient CD8^+^ T cells, numbers of TILs isolated from tumor tissue after 6 days increased substantially. Most significantly, PD‐1 and TIM‐3 surface expression was reduced on Nur77‐deficient CD8^+^ TILs and tumors were almost eradicated 25 days after their transfer. Thus, these experiments demonstrate that Nur77 is essential for T cell tolerance and represses antitumor immunity.

The second study concerned CAR T cells; such T cells are engineered to recognize specific antigens that are associated with distinct types of cancer and induce a pro‐inflammatory response against cancer cells carrying these antigens. Previous literature has shown that CAR T cells can efficiently target B‐cell malignancies [19, 20]. However, they are less effective against solid tumors because in the tumor microenvironment CAR T cells tend to exhaust, becoming dysfunctional. Chen et al. [14] transferred mouse CAR T cells targeting human (h)CD19 in mice bearing B16‐OVA‐hCD19 tumors and performed a detailed analysis of chromatin accessibility profiles. Both isolated CAR TILs and endogenous TILs showed enrichment for consensus NR4A motifs. In addition, the expression of NR4A members was higher in highly exhausted PD‐1^hi^ TIM3^hi^ TILs compared to “antigen‐specific memory precursor” PD‐1^hi^ TIM3^lo^ TILs. Detailed analysis of publicly available databases of single‐cell RNA sequencing from human CD8^+^ TILs also revealed a positive correlation between NR4A expression and exhaustion‐related gene expression of PD‐1 and TIM‐3 [21, 22]. Next, triple NR4A KO CAR T cells were transferred into mice with solid tumors leading to tumor eradication and an increased survival rate. Gene expression and chromatin accessibility profiles indicated increased effector function (*Il2*, *Ifng*) in triple NR4A KO compared to WT CAR TILs, which may provide the underlying mechanism. In addition, NR4As seemingly promote monomer NFAT DNA binding to exhaustion‐related target genes, PD‐1, and TIM3. On top of that, monomeric NFAT in turn promotes Nur77 transcription, indicative of a possible positive feedback [23]. Notably, genes involved in mitochondria metabolism were inhibited in Ttol cells[14], which is in accordance with previous studies suggesting an inhibitory role of Nur77 in mitochondrial metabolism in macrophages [24].

Taken together, the findings of these two novel studies indicate Nur77 (Nurr1 and NOR‐1) to be an important player in T cell tolerance and exhaustion in solid tumors. We deem it necessary to revisit knowledge of earlier literature to gain a deeper understanding on the precise function of Nur77 in T cells.

## Regulation of Nur77 transcription downstream of TCR signaling

**Figure 1:**
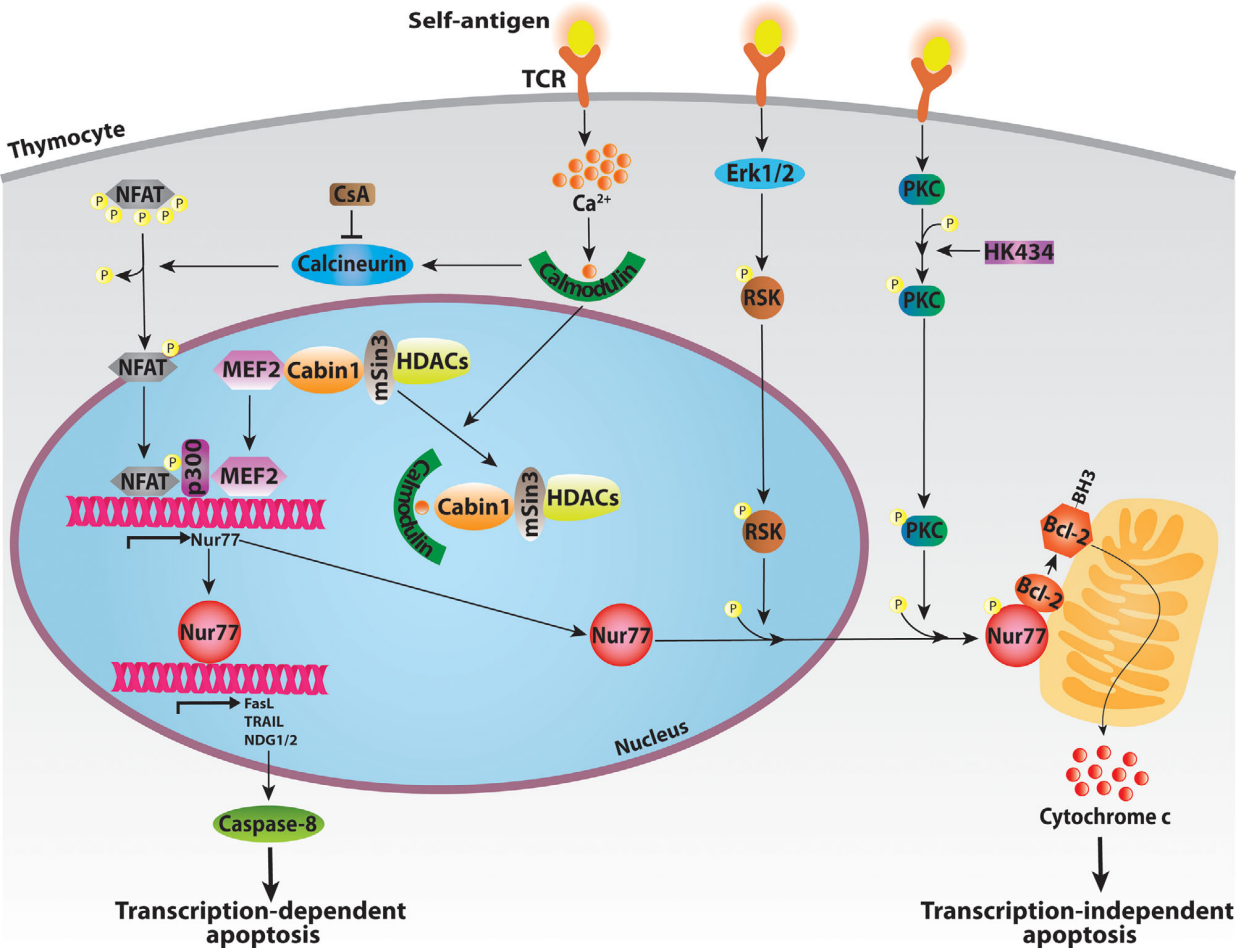
Regulation of Nur77 expression and activation of downstream pathways in thymocytes. TCR stimulation by self‐ligand binding increases intracellular calcium (Ca^2+^). Calcium activates calmodulin, which regulates Nur77 transcription in distinct ways. Calmodulin can form a complex with calcineurin, dephosphorylating nuclear factor of activated T‐cells (NFAT). NFAT then translocates to the nucleus, where it binds MEF2, enhancing its affinity for co‐activator p300, inducing Nur77 transcription. Alternatively, calmodulin releases MEF2 from Cabin1 by competitive binding. Once Nur77 is expressed it can directly bind promoters of its target genes, activating the transcription‐dependent apoptosis program. To activate transcription‐independent pathways, ERK1/2 and PKC are required, inducing translocation of Nur77 from the nucleus to mitochondria (involving protein phosphorylation). There, Nur77 interacts with Bcl‐2 proteins, exposing their BH3‐domain, inducing cytochrome c mediated organelle dysfunction and apoptosis.

Nur77 is an immediate‐early stress‐response gene induced in various tissues by stimuli such as pro‐inflammatory cytokines, hormones, and cell stress. In T cells, it is mostly induced by T cell receptor (TCR) activation [25, 26]. To assess the extent of TCR activation, Moran et al. [27] generated transgenic Nur77:GFP reporter mice, which express GFP under control of the endogenous Nur77 promoter on a bacterial artificial chromosome. In these mice, the strength of TCR signaling is accurately reflected by the intensity of the GFP signal in T cells [27]. In response to TCR triggering, Nur77 expression is rapidly induced, and this is dependent on an increased level of intracellular calcium and binding of calcium to calmodulin in the cytoplasm [28]. This in turn enhances Nur77 transcription through activation of the phosphatase calcineurin, which dephosphorylates the anti‐inflammatory NFAT. Dephosphorylated NFAT then translocate to the nucleus [29, 30], engage the transcription factor myocyte enhancer factor 2 (MEF2) that together with the coactivator p300 bind to MEF2 response elements in the Nur77 promotor and increase its expression [23, 31, 32]. Alternatively, calcium‐bound calmodulin can also activate the calmodulin‐dependent kinase type IV/Gr (CaMKIV/Gr) that initiates, a less well‐understood, distinct signal transduction pathway that induces Nur77 expression [31] (Fig. 1).

To decrease Nur77 transcription, a complex consisting of mSin3 (a transcription repressor protein), histone deacetylases 1 and 2, and the calcineurin‐binding protein cabin‐1 (Cabin1) attenuates MEF2 activity by limiting binding of the coactivator p300 to MEF2 [33]. As part of its Nur77‐inducing activity, calcium‐calmodulin can also translocate to the nucleus where it binds Cabin1 directly, which leads to the dissociation of Cabin1 from MEF2. This allows MEF2 to bind with p300 again, activating the Nur77 promoter [34]. Notably, consistent with the above‐described calcium‐dependent signaling cascade, inhibition of calcineurin by the immunosuppressive drug cyclosporin A (CsA) is known to inhibit Nur77 expression [25, 35].

Next to TCR signaling, Nur77 expression in T cells can also be regulated by retinoic acid (RA), a metabolite of vitamin A. RA is produced in the thymus to activate apoptosis in immature T cells, independent of TCR stimulation [36]. A study performed in mouse thymocytes showed that 9‐*cis*‐RA, a naturally occurring RA, activates the nuclear receptor RXR resulting in induction of Nur77 expression and Nur77‐dependent apoptosis [37, 38].

## Mechanisms of Nur77‐induced apoptosis

Apoptosis plays a pivotal role in development and selection of thymocytes and in T cell maturation. Nur77 modulates several downstream signaling pathways that culminate in apoptosis, in which both nuclear and mitochondrial pro‐apoptotic functions have been ascribed to Nur77 [39, 40, 41]. The ability of Nur77 to induce transcription‐independent apoptosis by directly promoting mitochondrial release of cytochrome c, is the best described pathway downstream of Nur77 (Fig. 1) [40]. This molecular mechanism involves regulation of the mitochondrial Bcl‐2 family of proteins, which generally serve as anti‐apoptotic factors [42, 43]. Bcl‐2 proteins carry at least one Bcl‐2 homology (BH) domain, of which the BH3 domain is characterized as pro‐apoptotic [43]. In cancer cells, binding of Nur77's LBD to Bcl‐2 has been shown to result in exposure of the BH3 domain and initiation of the apoptotic cascade [43, 44]. Similarly, TCR‐stimulation in thymocytes has been demonstrated to induce translocation of Nur77 from the nucleus to the mitochondria, thereby promoting Bcl‐2‐mediated thymocyte negative selection [45, 46].

A second mechanism underlying the pro‐apoptotic activity of Nur77 involves its interaction with protein kinase C (PKC) facilitating recruitment of Nur77 to the mitochondria [32, 47]. At the same time, Nur77 inhibits the kinase activity of PKC, which implicates that Nur77 may function in a negative feedback loop because PKC activates the transcription factor AP‐1 that is known to enhance Nur77 expression [48].

Third, ribosomal protein S6 kinase (RSK), activated by ERK1/2, phosphorylates Nur77 at serine 354 leading to Nur77 nuclear export (Fig. 1) [46]. Interestingly, ERK1/2 shares characteristics with Nur77, such as strong activation upon TCR stimulation, while its inhibition also leads to repressed apoptosis in thymocytes [49, 50]. This could imply that ERK1/2 facilitates Nur77 export from the nucleus to induce mitochondria‐involved apoptosis in thymocytes.

Finally, heterodimerization in the DBD interfaces of Nur77 and RXRα is known to accelerate translocation of this complex to the cytoplasm due to the presence of a nuclear export signal (NES) in RXRα [51]. It is interesting that 9‐cis‐RA, a known ligand of RXR, has been described to repress DBD‐mediated dimerization of RXRα and Nur77 using purified proteins. It can be speculated that this allows heterodimerization through the LBD which silences the NES of RXRα. However, this has not been shown in cells (or T cells) thus far, where 9‐*cis*‐RA only has been described to induce Nur77 expression [37].

Knowing Nur77 as a transcription factor, there is also a transcription‐dependent apoptotic pathway at play. Upon retinoid treatment, FasL, TRAIL, NDG‐1, Gpr65, and Bid were induced in a Nur77‐dependent way, and subsequently caspase 8 is activated (Fig. 1). Once activated, caspase 8 cleaves Bid, a BH3‐only Bcl‐2 protein located on the mitochondrial membrane, resulting in cytochrome c release [37, 52, 53]. In the same study, Nur77‐induced STAT‐1 expression was reported, which enhances Bim activity, another BH3‐only Bcl‐2 protein, leading again to cytochrome c release [37]. Microarray analysis of fetal thymus derived from WT and Nur77‐deficient mice revealed, in addition to NDG1, also NDG2 as a downstream Nur77 target gene [53]. NDG1 has a known function in apoptosis, but even though NDG2 targets mitochondria, its role in apoptosis remains to be defined [54]. So far, none of these apoptosis‐related genes have been demonstrated to be direct transcriptional targets of Nur77.

In contrast to these data, several studies reported no indications for the mitochondrial localization of Nur77, together with inconsistencies regarding the role of Bcl‐2 in Nur77‐induced apoptosis [53]. In line with these observations, interactions between Nur77 and Bcl‐2 could also not be detected in D011.10 T cells [45, 46, 53]. Because of the diametrically opposed outcomes of TCR stimulation with antigens, many of the results obtained will critically depend on the kind of co‐stimulation and differentiation states of the T cells used, so these kinds of contrasting findings may not be surprising.

## In vivo studies showing a role for Nur77 in thymocyte selection and mature T cell development

To date, most experiments in which the consequence of loss of Nur77 expression on the immune system and T cell function was studied were conducted in Nur77‐KO mice [55]. We and others have reported that aged Nur77‐KO mice develop immune cell infiltrates in the liver and enlarged spleens [56, 57, 58]. However, our recent study revealed that these mice unexpectedly express a truncated form of Nur77 that retains functional in stabilizing hypoxia induced factor in the BM, which may cause the liver and spleen anomalies [57]. To correct for this, we generated a new Nur77 model, based on the NR4A1‐floxed mice, and found that complete ablation of Nur77 does not result in the above‐mentioned phenotype. As such, we caution about interpretation of studies in which mice carrying the truncated Nur77 allele have been used and highlight the need to experimentally revisit the role of Nur77 in T cells in mice with a complete deletion of this gene.

Nur77 has been described to impinge on thymocyte selection [41]. Thymocytes undergo an intricate selection program in the thymus involving negative selection via apoptosis of non‐selected cells. Negative selection is promoted by high TCR stimulation of double positive (CD4^+^CD8^+^) thymocytes through binding of self‐antigens or self‐MHC molecules, which leads to high Nur77 protein expression [25]. Given that Nur77 specifically activates apoptosis in T cells, it thus may play an important role during negative selection of T cells [41]. Double‐positive thymocytes with low affinity to self‐antigens, and as a consequence with low Nur77 levels, go through positive selection and transition into single‐positive (CD4^+^CD8^−^ or CD4^−^CD8^+^) thymocytes. A small pool of double‐positive thymocytes with moderate Nur77 expression show upregulation of FoxP3 and thereby escape negative selection, differentiating into regulatory T cells (Treg cells) [59, 60], which will be described in detail below.

The discovery that Nur77 actively promotes negative selection of thymocytes was substantiated by an extensive number of mouse models. First, Nur77 deficiency in mice was repeatedly shown not to affect the number of immature CD4^−^CD8^−^, CD4^+^CD8^+^, and CD4^+^CD8^−^ thymocytes [55, 61]. Nowyhed et al. [61], however, did report an increase in CD4^−^CD8^+^ thymocytes. It should be taken in consideration though that the remaining NR4A members, Nurr1 and NOR‐1, are still expressed in these mice and may be functionally redundant. Therefore, transgenic mice were generated that express a dominant‐negative Nur77‐variant specifically in thymocytes under control of the *lck*‐promoter. In these mice, the transcriptional activity of all three NR4As is inhibited in thymocytes, resulting in reduction of apoptosis after TCR stimulation and increasing numbers of CD4^+^CD8^−^ thymocytes [32, 62]. Next, mice were generated with constitutively overexpressed Nur77 in thymocytes showing a large decrease in thymocytes, specifically immature CD4^+^CD8^+^, but also CD4^+^CD8^−^ and CD4^−^CD8^+^ thymocyte numbers were lower [63, 64]. The number of CD4^−^CD8^−^ thymocytes showed no difference when using mice with Nur77 overexpression in multiple studies [61, 63, 65], whereas one study observed an increase [66] and another lower numbers [64]. The inconsistent results obtained from these studies are most likely due to the use of different TCR transgenic backgrounds, type of T cells studied, and the stimulations applied in the accompanying in vitro experiments.

Mouse models have also been applied to assess the role of Nur77 in mature T cells. Similar to thymocytes, TCR stimulation in mature CD4^+^ and CD8^+^ T cells leads to Nur77 expression [66, 67]. In Nur77‐deficient mice, CD8^+^ T cells show an increased proliferation rate, and there are larger numbers of T helper type 1 (Th1) and Th17 cells [68, 69]. In addition, Nur77‐deficient mice show elevated CD69 and CD25 expression, suggesting increased activation of T cells [58]. Equally important, Nur77‐overexpression mice show a decrease in mature T cells in the spleen [64] and an increase in mature T cell apoptosis [66]. In addition, Nur77‐overexpression mice bear less peripheral semi‐invariant natural killer T (iNKT) cells due to high apoptotic rates [64]. Thus, impaired Nur77 expression promotes T cell activation and skews the delicate balance between proliferation and apoptosis.

To date, studies examining the roles of Nur77 in subsets of T cells, besides CD4^+^, CD8^+^, and iNKT cells have not been reported. In Nur77/Nurr1/NOR‐1 triple KO mice, CD4^+^ T cells in the periphery show accelerated differentiation into T helper type 2 (Th2) cells [70]. Single‐cell RNA sequencing of mouse Th17 cells isolated from lymph nodes and the CNS during autoimmune encephalomyelitis, or during in vitro differentiation revealed a subset of Th1‐like memory cells showing heightened Nur77 expression [71].

## Differential regulation of Nur77 activity in distinct T cell types

In contrast to thymocytes, mature T cells undergo activation through TCR signaling in combination with co‐stimulation, IL‐2 receptor signaling, and cytokines. This way, mature T cells are skewed toward expansion and specialized effector functions involving proliferation, clonal expansion, and differentiation. Therefore, TCR signaling in mature T cells mostly has a different outcome to TCR signaling in thymocytes. Both, in thymocytes and mature T cells Nur77 expression is induced upon TCR signaling [25], however, only in thymocytes does this induction lead to apoptosis in a Nur77‐dependent manner. In stimulated naïve mature T cells, the apoptotic role of induced Nur77 seems suppressed. Posttranslational modification in the form of phosphorylation has been described in T cells and will be discussed in this paragraph. We consider that co‐stimulation and cytokines affect Nur77 activity through differential phosphorylation.

**Figure 2:**
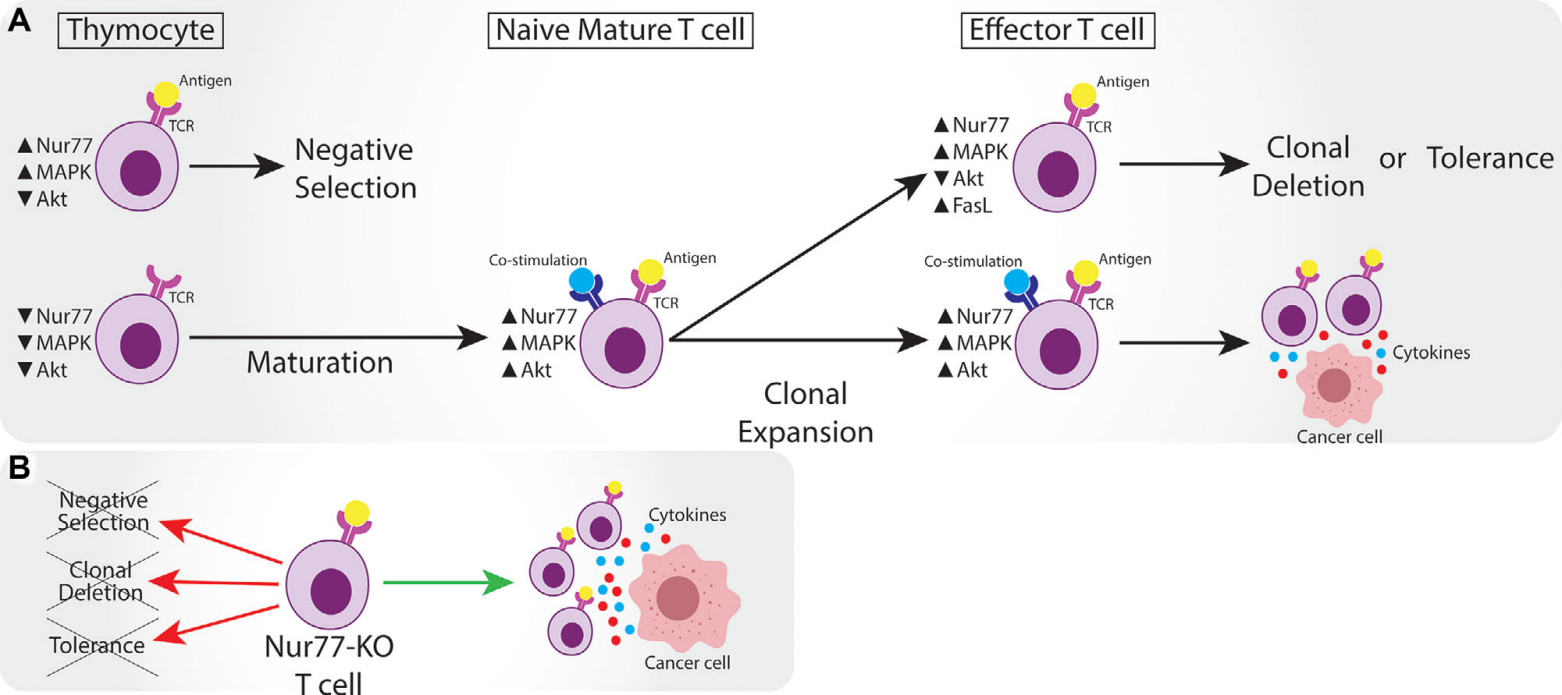
Overview of Nur77 expression in different subsets of T cells. A) Thymocytes that bind an antigen in the thymus upregulate Nur77 expression together with MAPK signaling, while Akt is lowly expressed, and undergo negative selection. Nur77, MAPK, and Akt expression remains low in thymocytes that do not bind antigen and then undergo maturation. Naïve mature T cells bind antigen and are co‐stimulated, leading to high Nur77 expression, but also high MAPK and Akt signaling that hyper‐phosphorylates Nur77. After clonal expansion, effector T cells can be continuously stimulated by TCR without co‐stimulation, leading to high Nur77 and MAPK, but low Akt expression. FasL (CD95L) upregulation leads to clonal deletion or tolerance/anergy. Effector T cells that are co‐stimulated preserve deactivated Nur77 through hyper‐phosphorylation highly express MAPK and Akt, thus able to target cancer cells and transcribe effector genes. B) Nur77‐KO T cells are resistant to negative selection and peripheral tolerance, defined by clonal deletion and tolerance (anergy). Nur77‐KO T cells become more efficient effector cells to target cancer.

In mature T cells, the two main signal transduction pathways are the MAPK/ERK‐ and PI3K/Akt‐pathway [72]. Both signaling pathways promote phosphorylation of Nur77, however do so on different serine residues; Ser354 and Ser350, respectively, resulting in distinct downstream effects [46, 73]. MAPK/ERK‐mediated phosphorylation of Nur77 is proapoptotic [46], while phosphorylation via the PI3K/Akt pathway promotes survival, migration, and differentiation [73, 74, 75] (Fig. 2). It is interesting to note that both ERK and Akt signaling are enhanced in activated thymocytes and mature T cells [67], but activation of Akt is more prominent in mature T cells [67, 76]. This is in line with the consensus that PI3K/Akt does not act as a binary (i.e., on/off) switch, but rather signals in a gradient manner [77]. Cunningham et al. [67] showed that Nur77 can be hyperphosphorylated (the exact amino acids phosphorylated were not determined) through crosstalk of MAPK/ERK‐ and PI3K/Akt‐pathways, keeping Nur77 inactive in the cytoplasm by preventing its translocation to both nucleus and mitochondria. However, it remains unclear what determines the exact magnitude of Akt activity in thymocytes and mature T‐cells. Inducible cell co‐stimulator (ICOS) [78] and polarization of lipid rafts stimulation sites [79], for instance, are known to activate the PI3K/Akt pathway in mature T cells and are not relevant in thymocytes.

Important to note, apoptosis does occur in mature T cells; for example, upon prolonged stimulation of effector T cells a large number of cells undergoes activation‐induced cell death (AICD) [72, 80]. There is an absence of co‐stimulation during AICD, therefore possibly preventing extensive upregulation of Akt to hyperphosphorylate Nur77 (Fig. 2). Besides this, continuous TCR stimulation leads to increased death receptor signaling, e.g., via CD95 (Fas), which is a transcriptional target of Nur77 [81] (Fig. 2). At present, there is no consensus regarding the mechanistic explanation for CD95 upregulation upon continuous TCR‐stimulation in AICD, except the absence of CD28 co‐activation [82].

Recently, it was shown that Nur77 controls development and self‐tolerance induction of iNKT cell [64]. TCR activation by agonistic self‐lipid ligands in developing thymocytes triggers iNKT cell lineage commitment, accompanied with an induction of Nur77 transcription in stage 0 iNKT cells [27]. Kumar et al. [64] used the Nur77‐overexpression mouse (*lck*‐promoter) on a specific TCR‐background (Vα14‐transgenic mice) to show that continuous expression of Nur77 decreases the number of iNKT cells in the thymus and periphery due to caspase‐3‐mediated apoptosis, similar as in conventional T cells. Specifically, Nur77‐overexpression leads to early developmental arrest in stage 0 iNKT cells. Besides inducing this negative selection of thymic iNKT precursors, sustained Nur77 expression promotes an exhausted and tolerant phenotype, recognized by high PD‐1 expression and a low cytokine response upon glycosphingolipid α‐galactosylceramide (αGC) stimulation [64]. Sustained Nur77 expression also impairs development of functional iNKT cells, such as NKT2, however the development of another functional subset, NKT17, remains unchanged. Kumar et al. propose that within different iNKT subsets distinct signaling pathways are triggered upon TCR stimulation. This leads to differential Nur77 regulation, resulting in a developmental difference of specific iNKT cell subsets. This suggestion is consistent with the above‐mentioned impact of Nur77 (hyper)phosphorylation on its downstream effect.

Together these data emphasize that posttranslational modification of Nur77 has a profound impact on its activity in thymocytes and well‐defined T cell subtypes. This is undoubtedly a subject that requires attention in future research, as is the need to assess the exact function of Nu77 in specific subsets of (effector) T cells.

## Nur77 enhances the suppressive capacity of T_regs_

Upon self‐recognition in the thymus, Nur77 expression in thymocytes evokes either negative selection or differentiation into Treg cells. The latter path is promoted by increased FoxP3 levels in CD4^+^ single‐positive thymocytes, which leads to a population of Treg cells in the periphery [60]. So far, the role of Nur77 in Treg development has remained elusive. Nevertheless, heterologous expression of Nur77 in mice resulted in elevated levels of FoxP3^+^ T cells in both the thymus and in secondary lymphoid tissue [64, 66, 68, 83]. Interestingly, once becoming CD4^+^FoxP3^+^, these Treg cells seem to be more resistant to Nur77‐induced apoptosis [66]. Given that high Akt signaling is known to have a negative effect on Treg development [84], PI3K/Akt signaling is most likely not the suppressor of Nur77 under this circumstance. Possibly, FoxP3 signaling pathways regulate Nur77 activity.

In a mouse model with all three NR4As specifically deleted in T cells, the emergence of Treg cells from CD4^+^ single‐positive thymocytes was prevented, and as a result the mice succumbed to systemic multiorgan autoimmunity [70]. These NR4A‐triple‐KO mice also had smaller populations of Treg cells in peripheral lymphoid organs as compared to WT mice [70]. Of note, data derived from NR4A‐triple‐KO mice preclude drawing conclusions on the specific role of Nur77 in Treg development and function. Another group demonstrated that Nur77‐deficient mice have a strong increase in FoxP3^+^ thymocytes, while no difference in Treg phenotype and CD25 or FoxP3 levels was reported [85]. The latter study suggests that Nur77 has a negative impact on Treg differentiation but not on how these cells function, as Nur77 promoted a Treg transcriptional signature, e.g., increased Ikzf2 and Tnfrsf9 expression [85]. These contrasting data may be due to distinct TCR transgenes that were applied in these studies that may have biased the extend of negative selection instead of Treg differentiation.

In the context of cancer, Treg‐mediated immune tolerance is important to consider. A large population of Treg cells is associated with poor prognosis in multiple types of human cancer, because these cells suppress the expansion of self‐reactive T cells [86]. Low numbers of self‐reactive T cells can influence immunological self‐tolerance against tumors, causing limited recognition of tumor cells. Accordingly, lowering the Treg population through inhibition or deletion of Nur77 has been shown to enhance detection of tumor cells without severe autoimmunity [87].

Taken together, Nur77 can influence the number of mature T cells directly through apoptosis in the thymus and indirectly by governing production of Treg cells that control expansion of mature T cells.

## Conclusion and perspective

After 30 years of studying Nur77 in T cell biology, this nuclear receptor turns out to affect not only thymocyte selection through apoptosis and the differentiation of mature T cells but also T cell tolerance and exhaustion in solid tumors (Fig. 2A). More specifically, recent studies revealed that high Nur77 expression in human TILs appears to correlate with high PD‐1 and TIM3 expression, both crucial components of the inhibitory immune checkpoint complexes. Moreover, in dedicated mouse models, deficiency of Nur77 (either together or not together with Nurr1 and NOR‐1) improves the effectiveness of TILs and CAR T cells in eradicating solid tumors [13]. On the other hand, Nur77 overexpression is known to function as an adequate therapeutic strategy to tackle auto‐immune disorders [88, 89].

Based on the most recent findings, inhibition of Nur77 in cellular cancer immunotherapy emerged as a serious option to improve the effectiveness of TIL preparations and CAR T cells. Even though it remains elusive how Nur77 precisely promotes tolerance and how this relates, if at all, to its apoptotic activity. In the end, both T cell anergy and apoptosis eventually result in peripheral tolerance and Nur77 seems to be a crucial player in both [90]. iNKT cells similarly show this phenomenon of self‐tolerance induction by Nur77, as Nur77 promotes both iNKT negative selection as well as induction of an exhausted phenotype resulting in iNKT cell anergy [64]. Future research should study the signaling pathways leading to anergy, and assess the exact role of Nur77.

Given that Nur77 has a strong anti‐inflammatory function both in the adaptive immune system and is protective in autoimmune diseases, major side effects may be expected when chronically inhibiting Nur77. More specifically, Nur77 has a beneficial influence on auto‐immune diseases [91], atherosclerosis [92, 93], MS [94], inflammatory bowel disease [13, 89], and inflammatory lung disease [95]. These diseases all worsen in Nur77‐deficient mice and involve chronic inflammation. Together these data illustrate that Nur77 is an important part of the intricate balance that maintains a properly functioning immune system protecting against infection, without causing inflammatory and auto‐immune diseases.

Based on this knowledge, targeting Nur77 in cellular immunotherapy is only a realistic option when executed with high specificity and in a transient manner. Small‐molecule NR4A antagonists may be best equipped to exert such function but are at present not available. The search for Nur77 antagonists is complicated by the mere fact that Nur77 has a non‐canonical LBD, lacking a traditional ligand‐binding pocket for which so far no high‐affinity endogenous ligand has been identified. As indicated in this review, posttranslational phosphorylation of Nur77 modulates the activity of this nuclear receptor, as well as homodimerization and heterodimerization with RXR, which may direct alternative drug design. Another important issue related to development of therapeutic strategies is the relatively unknown functional redundancy in T cell anergy of the three NR4A receptors: Nur77, Nurr1, and NOR‐1. Obviously, it will be necessary to assess how specific antagonists need to be for an optimal effect. Once antagonists have been identified it would be interesting to study whether Nur77 inhibition also improves non‐cellular immunotherapy involving inhibitors of immune checkpoint complexes, to limit tolerance and exhaustion of T cells.

In conclusion, Nur77 is downstream of TCR‐stimulation and will ultimately induce apoptosis or tolerance in T cells when activated. The only exception are Treg cells, which seem to thrive on Nur77 expression [66]. Overall, high levels of activated Nur77 leads to a decrease in thymocytes and mature T cells and induces tolerance and exhaustion in the remaining T cells, whereas deficiency (or inhibition) of Nur77 leads to an increase in T cell numbers and a strong immune response targeting tumors (Fig. 2B). As Nur77 seems one of the key players in the immunological balancing act, carefully inactivating it for an unbalanced attack on tumors could hold tremendous promise for the future.

## Conflict of interest

The authors declare no commercial or financial conflict of interests.

**Abbreviations**
AICD: activation‐induced cell death
CAR: chimeric antigen receptor
DBD: DNA‐binding domain
iNKT: semi‐invariant natural killer T cells
LBD: ligand‐binding domain
PD‐1: programmed death protein‐1
RXR: retinoid X receptor
TAD: trans‐activation domain
